# Transcriptomes Divergence of *Ricotia lunaria* Between the Two Micro-Climatic Divergent Slopes at “Evolution Canyon” I, Israel

**DOI:** 10.3389/fgene.2018.00506

**Published:** 2018-11-14

**Authors:** Chaoju Qian, Xia Yan, Hengxia Yin, Xingke Fan, Xiaoyue Yin, Peipei Sun, Zhijun Li, Eviatar Nevo, Xiao-Fei Ma

**Affiliations:** ^1^Key Laboratory of Stress Physiology and Ecology in Cold and Arid Regions, Department of Ecology and Agriculture Research, Northwest Institute of Eco-Environment and Resources, Chinese Academy of Sciences, Lanzhou, China; ^2^Key Laboratory of Ecohydrology of Inland River Basin, Northwest Institute of Eco-Environment and Resources, Chinese Academy of Sciences, Lanzhou, China; ^3^State Key Laboratory of Plateau Ecology and Agriculture, Qinghai University, Xining, China; ^4^College of Resources and Environment, University of Chinese Academy of Sciences, Beijing, China; ^5^Xinjiang Production & Construction Corps Key Laboratory of Protection and Utilization of Biological Resources in Tarim Basin, Alar, China; ^6^Institute of Evolution, University of Haifa, Haifa, Israel

**Keywords:** sympatric speciation, environment heterogeneity, *Ricotia lunaria*, genetic divergence, reproductive isolation

## Abstract

As one of the hotspot regions for sympatric speciation studies, Evolution Canyon (EC) became an ideal place for its high level of microclimatic divergence interslopes. In this study, to highlight the genetic mechanisms of sympatric speciation, phenotypic variation on flowering time and transcriptomic divergence were investigated between two ecotypes of *Ricotia lunaria*, which inhabit the opposite temperate and tropical slopes of EC I (Lower Nahal Oren, Mount Carmel, Israel) separated by 100 m at the bottom of the slopes. Growth chamber results showed that flowering time of the ecotype from south-facing slope population # 3 (SFS 3) was significantly 3 months ahead of the north-facing slope population # 5 (NFS 5). At the same floral development stage, transcriptome analysis showed that 1,064 unigenes were differentially expressed between the two ecotypes, which enriched in the four main pathways involved in abiotic and/or biotic stresses responses, including flavonoid biosynthesis, *α*-linolenic acid metabolism, plant–pathogen interaction and linoleic acid metabolism. Furthermore, based on *Ka*/*Ks* analysis, nine genes were suggested to be involved in the ecological divergence between the two ecotypes, whose homologs functioned in RNA editing, ABA signaling, photoprotective response, chloroplasts protein-conducting channel, and carbohydrate metabolism in *Arabidopsis thaliana*. Among them, four genes, namely, *SPDS1, FCLY, Tic21* and *BGLU25*, also showed adaptive divergence between *R. lunaria* and *A. thaliana*, suggesting that these genes could play an important role in plant speciation, at least in Brassicaceae. Based on results of both the phenotype of flowering time and comparative transcriptome, we hypothesize that, after long-time local adaptations to their interslope microclimatic environments, the molecular functions of these nine genes could have been diverged between the two ecotypes. They might differentially regulate the expression of the downstream genes and pathways that are involved in the interslope abiotic stresses, which could further diverge the flowering time between the two ecotypes, and finally induce the reproductive isolation establishment by natural selection overruling interslope gene flow, promoting sympatric speciation.

## Introduction

Sympatric speciation, first proposed by Darwin as speciation occurring in contiguous populations with ongoing gene flow, was considered as one of the important models of biodiversity origin (Darwin, 1859). Compared to other geographic models of speciation, such as the allopatric and parapatric speciation models, sympatric speciation attracts particular interests for the reproductive barriers must evolve *in situ* to prevent homogenization, which was challenging to be proved between contiguous populations (Mayr, 1947, 1963; Fitzpatrick et al., 2008; Bird et al., 2012). Previous empirical studies and theoretical modeling supported that sympatric speciation was possible (Smith, 1966; Gavriletz, 2004; Bolnick and Fitzpatrick, 2007). However, it required the conditions of primary divergence with gene flow between populations, with complete absence of geographic barriers, which could be uncommon in nature. Recently, a plenty of studies on animal and bacteria revealed that the formation of reproductive isolation during the sympatric speciation could be favored by natural selection, such as in sexual selection and resource competence (Berlocher and Feder, 2002; Li K. et al., 2015, 2016; Rosser et al., 2015; Dunning et al., 2016; Getz et al., 2016; Kautt et al., 2016; Rodriguez et al., 2016; Zhao et al., 2016). Up to date, however, there is currently very little evidence to prove that natural selection in plants could induce sympatric speciation, partially due to the fact that flowering time hardly diverged between contiguous populations.

With high environmental heterogeneity, mountains supply diversifying habitats for biomes, which could have promoted the sympatric speciation. As one of the most promising models to address the sympatric speciation, “Evolution Canyon” (EC I) (Lower Nahal Oren, Mount Carmel, Israel) supplied plenty of cases in sympatric speciation across life from bacteria to mammals, which showed that similar genomic and phenotypic differentiation across species occurred between microsites with high microclimatical heterogeneity (Nevo, 2009). Such microclimatic differentiation has considerable consequences in population genetic structure within diverse organisms (Nevo, 1997, 2012; Rashkovetsky et al., 2006; Hadid et al., 2013, 2014), and the EC model reveals interslope divergence across life from bacteria to plants and mammals, followed by divergent genomic, proteomic, and phenomic adaptive complexes (Nevo, 2012, 2014, 2015; Hadid et al., 2014; Buse et al., 2015; Finkel et al., 2015). In the EC, major adaptive complexes on the tropical “African” slope [(AS), also called south-facing slope (SFS)] are related to solar radiation, heat, and drought, whereas those on the temperate “European” slope [(ES), also called north-facing slope (NFS)] are related to shade stress for photosynthesis, and interslope species divergence led to the incipient sympatric speciation in a diversity of organisms (Pavlícek et al., 2003; Nevo, 2006, 2014; Kossover et al., 2009; Yang et al., 2009; Li K. et al., 2016). This kind of local adaptation resulted from interslope microclimatic divergence also occurring in other three “Evolution Canyons” in Israel in the Galilee, Golan, and Negev Mountains as well as in the other evolution canyons worldwide (Nevo, 2012), and in the extension of the Evolution Canyon model in Evolution Plateau (upper Galilee Mountains) (Hadid et al., 2013; Li K. et al., 2015, 2016; Zhao et al., 2016).

Among the diverse organisms, an annual plant species of Brassicaceae (Warwick and Al-Shehbaz, 2006), *Ricotia lunaria* inhabits both contrasting slopes of EC I. Based on the AFLP (amplified fragment length polymorphism) and full genome array hybridizations in *R. lunaria* from the contrasting opposite slopes of EC I, previous studies showed that both the genetic diversity and transcriptome expression pattern differed significantly between interslope ecotypes. Up-regulation of drought resistance genes were witnessed in the SFS individuals, while the photosynthetic genes were upregulated in the NFS individuals, suggesting that the expression patterns diverged between interslopes due to environmental heterogeneity (Brodsky et al., 2008; Kossover et al., 2009). Unfortunately, little is known about how the microclimatic heterogeneity affected the phenotypic differentiation and even the genomic divergence between ecotypes from the two slopes. On the other hand, *R. lunaria* has relatively big and heavy seeds, and the gravity should play a significant role in its seed dispersal, suggesting higher intraslope than interslope gene flow. This supported the observation of higher genetic divergent interslopes than intraslopes (Kossover et al., 2009). For bottom slope populations at EC I, however, gravity did not intensify the gene flow between the slopes. Thus, the phenotypic and genetic divergence between the two bottom contrasting slope populations (#3 on SFS and #5 on NFS), could be better explained by microclimatic heterogeneity between the SFS and NFS.

**FIGURE 1:**
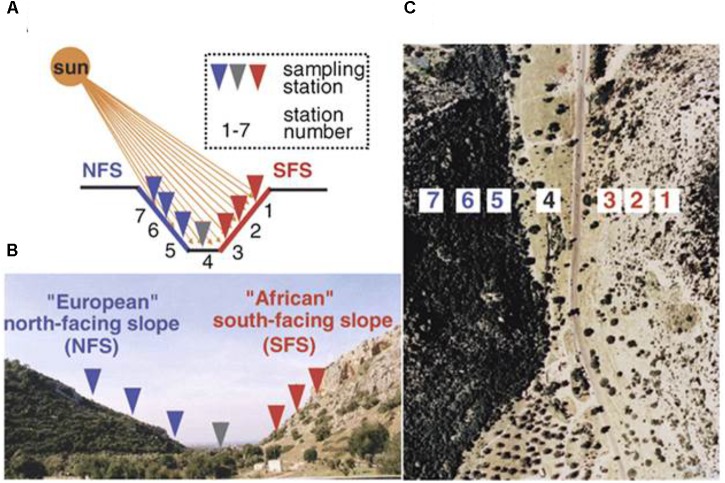
The opposing slopes of “Evolution Canyon” I, Lower Nahal Oren, Mount Carmel. The tropical xeric “African” slope is on the right, and the temperate mesic “European” slope is on the left. Panel **(A)** is the schematic diagram, panel **(B)** is the cross section view of EC I, Lower Nahal Oren, Mount Carmel, and panel **(C)** is the air view of EC I (Nevo, 2012), only one genotype from the station 5 and one genotype from the station 3 were analyzed in this study.

In this study, we planted two ecotypes of *R. lunaria* from the bottom populations of the opposite slopes (population #3 on the dry tropical savannoid SFS and population #5 on the humid temperate forested NFS from EC I) (Figure 1). We investigated both the phenotypic variations of flowering time and the transcriptomic expression patterns between them. We aimed to address the following questions of adaptive interslopes micro-ecological divergence across ∼100 m: (1) whether the flowering times were significantly differentiated between the two interslope ecotypes; (2) whether the expression or sequence variations on the flowering time genes were also diverged between the two ecotypes? Likewise, what regulatory modules of genes or pathways that are adapted to the interslope microclimatic divergence induce the differentiation of flowering time between the two ecotypes? The investigation on both phenotypic and transcriptomic divergence of these two ecotypes of *R. lunaria*, will shed light on our better understanding of the molecular basis of sympatric speciation in plants in ecologically divergent microsites.

## Materials and Methods

### Plant Materials and Physiological Measurements

Two ecotypes of *R. lunaria* seeds were collected in the year 2012. As shown in Figure 1, one was from station 3 (SFS3) of EC I under savannoid tropical high illuminance, high temperature and dry growing conditions. By contrast, another one from the forested temperate opposite slope of EC I station 5 (NFS5), under the opposite growing conditions to the SFS3 ecotype with shade and humid environment. Before planting, the seeds were stored for 1 month at -20°C to synchronize their germinations. To verify whether genes under stress were constructively differently expressed between the two interslope ecotypes, five replicates for both the two ecotypes were planted in the same growth chamber at 23°C under long day conditions of 18 h light and 6 h dark, with 53% humidity conditions. The flowering times of the two ecotypes were recorded during the plants’ growth. Different tissues of the two ecotypes were collected for RNA isolation at the stage of the first flowering, and all materials were sampled at 10 am and immediately frozen in liquid nitrogen.

### RNA Extraction, cDNA Library Preparation, and RNA-seq

Total RNA samples from root, stem, leaf, and bud for each ecotype were extracted using the E.Z.N.A. Plant RNA Kit (Omega Bio-tek, United States) with 2% PVBB added, and the remaining DNA was removed by RNase-free DNase (Omega Bio-tek, United States) according to the instruction manual. RNA concentration and purity of each sample was determined with ratio of OD260/280 by NanoDrop2000™ micro-volume spectrophotometer (Thermo Scientific, Waltham, MA, United States), and all the samples passed quality control as the ratio of OD260/280 between 1.9 and 2.2 and the ratio of OD260/230 was less than 2.0. Then, the RNA integrity was further verified by 1.5% agarose gel electrophoresis with two clear bands of 28S/18S ribosomal RNA. Total RNA samples for each ecotype was pooled together including the root, stem, leaf, and bud in the ratio of 1:1:1:2 to construct the cDNA library, respectively.

The poly-A mRNA was enriched with magnetic Oligo (dT) beads and then fragmented into short fragments as the templates to synthesize the cDNA fragments as detailed by Shi et al. (2013). The cDNA fragments were further purified with a QiaQuick PCR extraction kit (QIAGEN Inc., Valencia, CA, United States), followed by end repairing and tailing A, and then were ligated to sequencing adaptors. The required length ligation products with 200–700 bp were purified by agarose gel electrophoresis and enriched by PCR amplification. Finally, the paired-end library was sequenced by the Illumina HiSeqTM 2000 platform with the average read length of 90 bp.

### *De novo* Assembly and Expression Profiling

After filtering adapter sequences, reads with ambigous sequences ‘N’ over 5% and reads with a base quality less than Q20, a total of 39.90 and 31.16 million clean reads were generated for NFS5 ecotype and SFS3 ecotype, respectively (Table 1). Then, Trinity was used to *de novo* assembly the high-quality clean reads into contigs (Grabherr et al., 2011). Short contigs were then clustered and assembled into the longest contigs with gaps according to the pair-end information and sequence similarity. The longest contigs in each cluster and singleton were combined together as the total unigenes. The RNA-seq raw data was deposited in the NCBI with SRA accession number of SRP150700.

To determine the unigene expression profiles, RPKM (reads per kilobases per million reads) of each unigene was computed by ERANGE3.1 software (Mortazavi et al., 2008). IDEG6 software (Romualdi et al., 2003) was also used to further identify the differentially expressed genes (DEGs) by pairwise comparison of the two ecotypes, with *p*-value < 0.01 and FDR significance score < 0.001 (Romualdi et al., 2003).

### Gene Structure and Functional Annotation

The ORF (open reading frame) for each unigene was predicted by the Getorf program of the EMBOSS software package (Rice et al., 2000). SNPs between the two ecotypes were detected using SOAPsnp^1^ with the default parameters (Li et al., 2008). Functional annotation of each unigene was performed by aligning with public protein and/or nucleotide databases (such as the NCBI Nr, Nt databases, Swiss-Prot protein database, COG database, and the KEGG database) by BLASTx with an E-value cutoff of 1e-5. Further annotation analysis, such as the biological process, molecular functions and cellular components, were also performed with GO terms by Blast2GO software (Conesa et al., 2005; Conesa and Gotz, 2008).

### Comparative Transcriptome Analysis and Putative Candidate Genes Involved in the Constructive Divergence of the Two Ecotypes

To further explore whether the constitute expression were divergent between the two ecotypes, differential expression analysis was first performed with EBSeq (Leng et al., 2013) under 1% false discover cutoff and at least twofold change. KEGG pathway analysis was also implemented to unravel the differential expression of genotype-dependent stress-responsive transcriptome pathways.

Based on SOAPsnp^1^, SNP calling was also performed between the transcriptomes of the two ecotypes to discover SNPs between the two genomes. Noteworthy, the whole-genome duplications in this genus (Lysak et al., 2007) could exaggerate the real number of the SNPs, as a large percentage of them could be due to the divergence between paralogous genes and unequal expression between the two ecotypes. Thus, the criteria were re-considered to guarantee as much as to further screen the SNPs on the orthologous genes, which were verified by the homologous unigene sequences based on very strict reciprocal localBlast, and then each of the unigene pairs were then trimmed into CDS fragments with SNPs.

We further identified the orthologous sequences with BLASTx against the unigenes library for each ecotype with *Arabidopsis thaliana* protein and/or nucleotide sequences from TAIR 11th public release ^2^ under the threshold of E-value ≤ 1e-5. Furthermore, to screen whether the homologous genes diverged adaptively between the two ecotypes in response to their local environments between the slopes, intra-species (*Pi*a/*Pi*s) within the two ecotypes of *R. lunaria* and inter-species divergence (*K*a/*K*s) between *R. lunaria* and *A. thaliana* for each homologous genes were estimated with KaKs_Calculator 2.0 (Wang et al., 2010) under the YN model of approximate method (Yang and Nielsen, 2000).

## Results and Discussion

### Transcriptome Variations of the Two *R. lunaria* Ecotypes

Two transcriptomes with total RNA samples combined from bud, leaf, stem, and root (with the ratio of 2:1:1:1) were sequenced. In total, over 14 Gbp clean data from 71 million reads were sequenced and passed the Q20 quality control, with 8 Gbp for the ecotype of NFS5 and 6.2 Gbp for the ecotype of SFS3, respectively (Table 1). Interestingly, the SFS3 ecotype had slightly higher, 0.69%, GC content than that of the NFS5 ecotype (Table 1), which suggests that long-term higher solar radiation, heat, and drought stress could have induced the genome of *R. lunaria* to evolve adaptively to be more stable in the south-facing slope than the opposite slope. This was widely found in bacteria from the interslopes, such as *Bacillus subtilis* (Barash et al., 2006; Nevo, 2012), and *Hallobacterium* species (Kennedy et al., 2001).

**Table 1:** Information of transcriptomes and flowering time of the two genotypes from the contrasting slopes.

Samples	BMK-ID	Total reads	Total nucleotides (bp)	Cycle Q20	GC contents	Date of sewing	First flowering	Time to flowering (days)
NFS5	T1	39,903,639	8,059,807,425	100.00%	46.62%	3rd July, 2012	30th November	149
SFS3	T2	31,160,621	6,293,935,181	100.00%	47.31%	3rd July, 2012	30th August	57

A total of 36,705 and 33,499 unigenes were assembled in the transcriptome of NFS5 ecotype and SFS3 ecotype, respectively. Both ecotype transcriptomes had N50 over 1,500 bp with the average length of 900 bp (Table 2). 47,103 unigenes were assembled when these two transcriptomes were pooled together, among which 14,341 (30.45%) unigenes were longer than 1 kb, suggesting the goodness of this *de novo* assembly. However, only 19,051 unigenes (40%) were shared with each other, while 16,163 unigenes were specific to NFS5 and 11,189 unigenes specific to SFS3. This phenomenon could have resulted for two reasons: (1) the one is for the vast divergence between the two ecotypes, which made it difficult to match the short reads together, since allelic SNPs and indels could introduce more isoforms by Trinity assembly (Grabherr et al., 2011); (2) the other one is for the incomplete sequencing of transcriptomes for both the two ecotypes.

**Table 2:** Length distribution of the two transcriptomes of *R. lunaria*.

Length range	NFS5 (T1)	SFS3 (T2)	All unigenes
200–300	9,434 (25.70%)	7,918 (23.64%)	12,372 (26.26%)
300–500	8,511 (23.19%)	7,539 (22.50%)	11,315 (24.02%)
500–1000	7,091 (19.32%)	7,065 (21.09%)	9,075 (19.26%)
1000–2000	7,367 (20.07%)	7,408 (22.11%)	9,058 (19.23%)
2000+	4,302 (11.72%)	3,569 (10.65%)	5,283 (11.21%)
Total number	36705	33,499	47103
Total length	34,223,242	30,845,185	42,725,664
N50 length	1,612	1,505	1,581
Mean length	932.39	920.78	907.07

**FIGURE 2:**
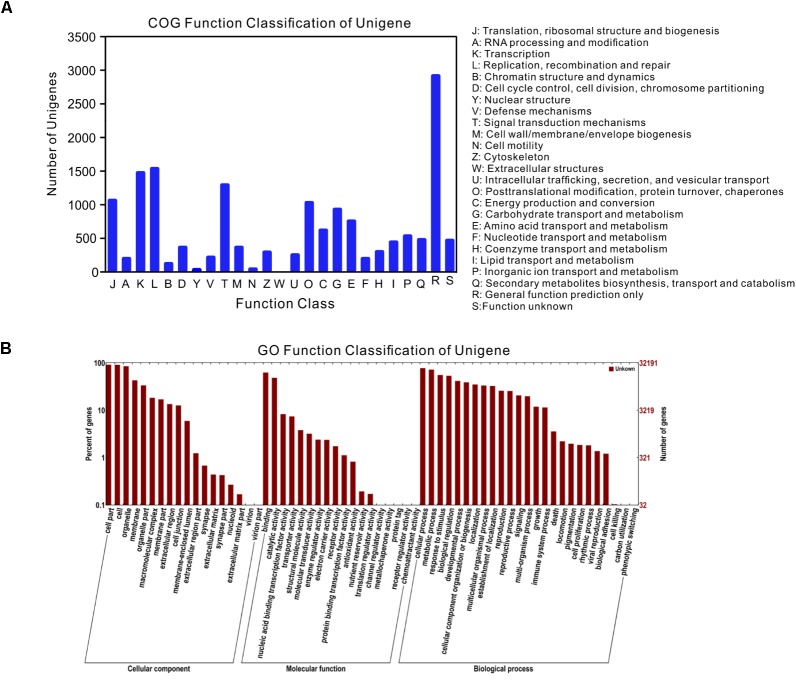
Gene annotations of unigenes. **(A)** Histogram presentation of clusters of orthologous groups (COG) classification, **(B)** good hits were aligned to the GO database.

Among the 47,103 unigenes, a total of 39,860 unigenes (84.6%, Table 3) could be annotated against with the public databases. Among these annotated unigenes, 34,878 unigenes (79.3%) can find the homologs in the Nt database with E-value ≤ 1e-5 (Table 3), and 10,559 unigenes out of them were classified into 24 COG categories (Figure 2A). Among these categories, the largest group is the “general function prediction only” (2,901, 27.47%), followed by “replication, recombination and repair” (1,511, 14.31%), “transcription” (1,449, 13.72%) and “signal transduction mechanisms” (1,266, 11.99%) (Figure 2A). A total of 32,191 unigenes were assigned into 10,942 GO terms of three main categories (cellular component, molecular function, and biological process) and 52 sub-categories (Figure 2B). Pathway-based analysis showed that 8,016 unigenes were assigned into 170 KEGG pathways, with the most highly represented category of “ribosome” (462 genes), “plant hormone signal transduction” (325 genes) and “oxidative phosphorylation” (287 genes) (Supplementary Table S1). Notably, most abundant genes in both of the two ecotypes were involved in oxidoreductase activity, photosynthesis, light harvesting, energy metabolism and defense responses to abiotic stress (Supplementary Table S2), which supported the hypothesis that environmental conditions in the growth chamber may be stressed for both ecotypes, especially for the light conditions, such as affecting population # 5 growing in shade.

### Constructive Differently Expressed Genes Between the Two Ecotypes in the Same Growth Chamber

**FIGURE 3:**
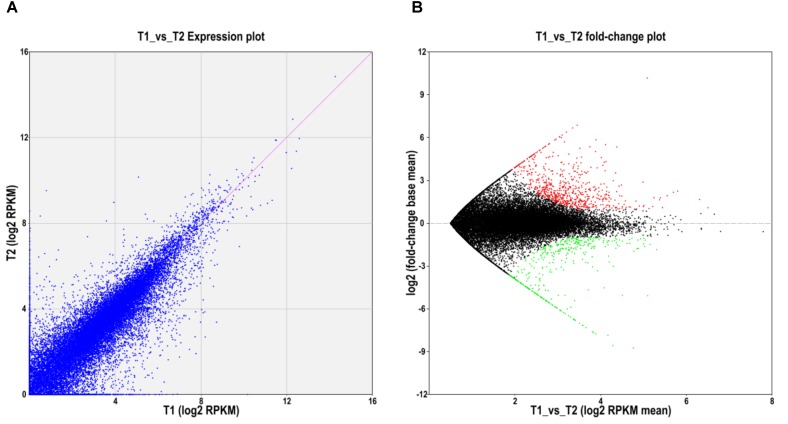
Differentially expressed unigenes between NFS5 (T1) and SFS (T2). **(A)** Expression abundance comparison; **(B)** Volcano plot for the unigenes expressed differently between the two ecotypes. Red dots represented that unigenes with the expression level significantly higher in NFS5 (T1), while green dots for that of higher ones in SFS3 (T2).

**FIGURE 4:**
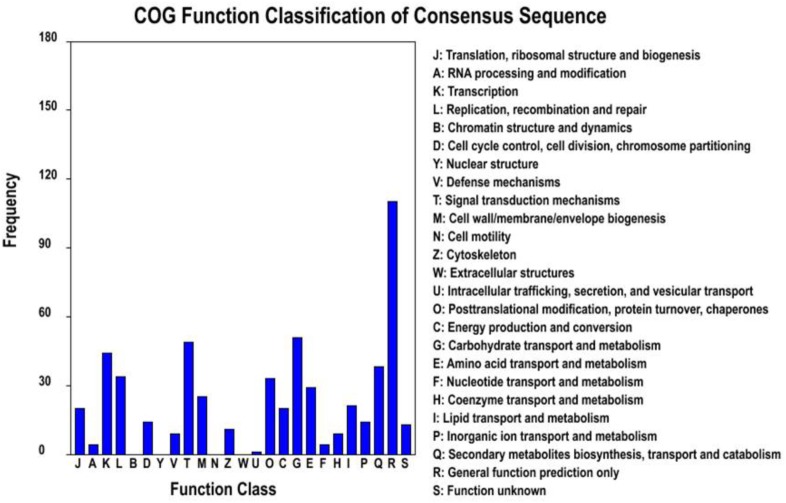
Histogram presentation of clusters of orthologous groups (COG) classification for DEGs.

To further explore whether the constitute expression was divergent between the two ecotypes, differential expression analysis was first performed with EBSeq (Leng et al., 2013) under 1% false discover cutoff and at least twofold change. Compared with the level of gene expression in SFS3 ecotype, a total of 1,064 unigenes were differentially expressed in the NFS5 ecotype, including 683 up-regulated and 381 down-regulated unigenes (Figure 3). Among the differentially expressed unigenes (DEGs), 553 unigenes were categorized in 21 COG clusters (E-value ≤ 1e-5) with the following five largest categories: general function prediction only (110 unigenes), carbohydrate transport and metabolism (51 unigenes), signal transduction mechanisms (49 unigenes), transcription (44 unigenes), and secondary metabolites biosynthesis, transport, and catabolism (38 unigenes) (Figure 4). Using the hypergeometric test compared to the genomic background, we further identified 42 significantly enriched GO terms of DEGs containing 976 unigenes (*p* ≤ 0.05, after Bonferroni correction, Supplementary Table S4), and all the top DEGs came from the pathways involved in biotic and abiotic responses, such as jasmonic acid biosynthetic process (GO:0009695), response to chitin (GO:0010200), response to fungus (GO:0009620), response to jasmonic acid stimulus (GO:0009753), response to mechanical stimulus (GO:0009612), abscisic acid mediated signaling pathway (GO:0009738), response to water deprivation (GO:0009414), and hyperosmotic salinity response (GO:0042538) (all the *p*-values as 0, Supplementary Table S4). Most of these processes were played a dominant role in stress defense for almost all the plant species analyzed, such as the jasmonic acid biosynthetic process (Robertseilaniantz et al., 2011; Pieterse et al., 2012).

**Table 3:** Summary of gene annotation against the seven databases.

Anno_Database	Annotated_Number	300 < = length < 1000	Length > = 1000
KEGG_Annotation	8,016	3,257	3,487
COG_Annotation	10,559	3,334	6,306
GO_Annotation	32,191	13,743	13,796
Swissprot_Annotation	26,829	10,635	12,521
TrEMBL_Annotation	34,830	15,177	14,141
Nr_Annotation	34,878	15,192	14,146
Nt_Annotation	37,285	15,814	14,025
All_Annotated	39,860	17,386	14,214

### Differential Expression Pathways Analysis

Results of differential expression analysis implemented with KEGG pathway analysis showed that after genomic abundance correction (Supplementary Table S3), four pathways showed significantly different expression between the two ecotypes: flavonoid biosynthesis (10 unigenes, with 7 unigenes up-regulated in ecotype NFS5, while the other 3 unigenes up-regulated in ecotype SFS3, *p* = 7.13 E-06, Supplementary Figure S1), *α*-linolenic acid metabolism (14 unigenes, with 7 unigenes up-regulated in ecotype NFS5, and the other 7 unigenes up-regulated in ecotype SFS3, *p* = 1.77 E-05, Supplementary Figure S2), plant–pathogen interaction (26 unigenes, with 14 unigenes up-regulated in ecotype NFS5, while the other 12 unigenes up-regulated in ecotype SFS3, *p* = 2.59 E-04, Supplementary Figure S3) and linoleic acid metabolism (6 unigenes, with 3 unigenes up-regulated in ecotype NFS5, and another 3 unigenes up-regulated in ecotype SFS3, *p* = 1.3 E-02, Supplementary Figure S4). Flavonoid were reported as an important group of secondary metabolites, which played an important role in plant growth, development, and reproduction. It was also involved in the responses to diverse abiotic stresses, such as the UV radiation, high light, low availability of water and nutrients, temperature fluctuations and even the pathogen infection (Lenka et al., 2011; Ferreyra et al., 2012; Pandey et al., 2016; Singh et al., 2017). This kind of different expression of flavonoid biosynthesis pathway between different ecotypes were also found in the other model plants responding to diverse environment factors (Azuma et al., 2012; Guidi et al., 2016). In this study, several important enzymes in the flavonoid biosynthetic pathway were also differentially expressed between the two ecotypes (Supplementary Figure S1), suggesting that it could be an effective response to various stress-induced injuries in the growth chamber for *R. lunaria*, such as in light or shade stress for each ecotype. The two major unsaturated fatty acids in membrane lipids of plant leaves, *α*-linolenic and linoleic acid, also contribute to plant defense responses for abiotic and/or biotic stresses as documented in other plant species (Li Q. et al., 2016; Lim et al., 2017). The plant–pathogen interaction pathway was also reported to be not only responsive to the biotic stresses but also to the abiotic stresses in plants (Dodds and Rathjen, 2010; Barakate and Stephens, 2016; Wang et al., 2017). These four pathways were also widely involved in various plant resistance against diverse abiotic and/or biotic stress-induced injuries, such as in drought, UV, and/or salt responses for peach (Li X.W. et al., 2015), apple (Yin et al., 2016), rice (Lenka et al., 2011), *Chorispora bungeana* and *Arabidopsis* (Zhao et al., 2012). To respond to the ecological stresses in the growth chamber, *R. lunaria* could rely on the integrated effects of different pathways, and these up-regulated enzymes involved in these pathways could also provide targets for further studies of the molecular regulation mechanism in response to specific environmental stresses, such as the key enzymes of DFR, OPCL1, and JAZ. Clearly, the common garden experiment in the growth chamber would highlight ecotype divergence.

### Genomic Divergence Between the Two Ecotypes

A total of 20,130 putative SNPs with scores over 30 were discovered in 6,107 genes. After filtering with strict reciprocal localBlast to avoid the paralogs, a total of 15,402 SNPs were finally found in 1,140 homologous genes between the two ecotypes, covering more than one million basepairs of the genome sequences (1.71 Mbp, with length varied from 164 bp to 6 kbp).

**FIGURE 5:**
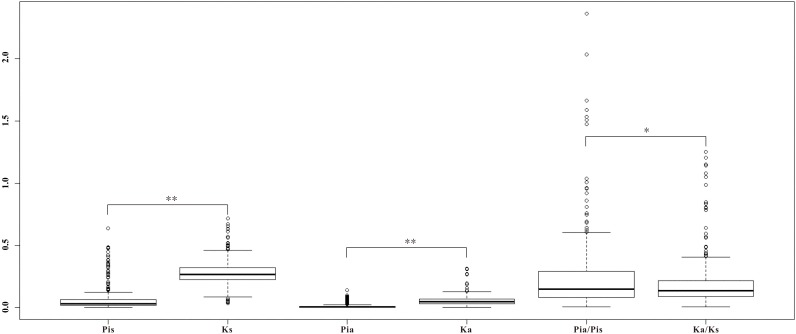
Boxplot of the divergence on the 281 homologs within *Ricotia lunaria* (Pia, Pis, and Pia/Pis) and between *R. lunaria* and *Arabidopsis thaliana* (Ka, Ks, and Ka/Ks). ^∗^*p*-value < 0.05, ^∗∗^*p*-value < 0.01, which was estimated with Student’s *t*-test.

To further investigate whether the homologous genes diverged between the two ecotypes responding to the interslope divergent environments, among the 1,140 homologous genes with SNPs, we identified a set of 281 homologous genes with both synonymous and non-synonymous mutations (Figure 5). As shown in Table 4, nine pairs of homologous genes were putatively involved in the adaptive ecotypes’ divergence from each other with the *Pi*a/*Pi*s ratio over 1: *T1_Unigene27281* vs. *T2_Unigene22713* (RNA recognition motif-containing protein), *T2_Unigene19717* vs. *T1_Unigene31973* (WPP domain-interacting protein 3), *T2_Unigene22075* vs. *T1_Unigene22516* (*spermidine synthase 1*), *T1_Unigene14300* vs. *T2_Unigene15331* (*regulatory component of ABA receptor 1*), *T1_Unigene30289* vs. *T2_Unigene25344* (*repressor of silencing 3*), *T1_Unigene25303* vs. *T2_Unigene21895* (*farnesylcysteine lyase*), *T1_Unigene24651* vs. *T2_Unigene25310* (*ZML2, GATA transcription factor 28*), *T1_Unigene24978* vs. *T2_Unigene23451* (*Translocon at inner membrane of chloroplasts 21*), and *T1_Unigene31300* vs. *T2_Unigene16505* (*BGLU25 beta glucosidase 25*). Based on previous functional annotations, we found that most of these divergent genes were involved in the post-transcriptional regulation, ABA-dependent response, photoprotective response, chloroplast protein-conducting channel, carbohydrate metabolic, and so on. For examples, *zinc finger protein expressed in inflorescence meristem like2* (*ZML2*), a GATA transcription factor, takes part in the photoprotective response mediated by the photoreceptor Cryptochrome 1 (cry1) (Shaikhali et al., 2012), which is a critical gene regulating flowering time in many plants (Yuan et al., 2016). *ZML2* also plays a role in wound-induced lignification as a transcriptional repressor for the lignin biosynthesis in maize (Velezbermudez et al., 2015). The *RNA recognition motif-containing* (*RRM*), one of the largest families of RNA binding proteins gene widely detected not only in plants and animals, but also in fungus, plays an important role in RNA recognition and editing (Fetka et al., 2000; Rebay et al., 2000; Samarajeewa et al., 2017). In plants, as documented in model plants of *Arabidopsis* and maize, *RRM* is required for plastid and mitochondrial RNA editing (Sun et al., 2013), and previous studies showed that an organelle *RRM* mutant exhibits slower growth and delayed flowering time (Shi et al., 2016). As most of these divergent genes were located in the up-stream of the biological pathways, we speculated that the adaptive divergence could rely more on sensing of multiple stresses with upstream regulations than with downstream regulations. To verify this hypothesis, of course, more transgenic experiments on these divergent alleles are still needed.

**Table 4:** Inter-species and intra-species divergence of genes involved into the ecological adaptation.

Gene ID	RPK M1	Homolog	RPK M2	Atgene	Gene annotation	La	Pi(s)	Pi(a)	Pi(a)/ Pi(s)	Ks	Ka	Ka/Ks	KEGG pathway
*T1_Unigene27281*	8.7	*T2_Unigene22713*	13.5	*At3G20930*	RNA recognition motif-containing	992	0.00444	0.01046	2.365	0.2553	0.08303	0.282	RNA editing
*T2_Unigene19717*	11.6	*T1_Unigene31973*	7.8	*At3G13360*	WIP3 WPP domain-interacting protein 3	610	0.00818	0.01654	2.034	0.17651	0.1187	0.642	
*T2_Unigene22075*	41.7	*T1_Unigene22516*	35.6	*At1G23820*	SPDS1 spermidine synthase 1	329	0.08724	0.13941	1.663	0.08724	0.09875	1.142	Glutathione metabolism
*T1_Unigene14300*	27.0	*T2_Unigene15331*	9.9	*At1G01360*	RCAR1 regulatory component of ABA receptor 1	534	0.00519	0.00822	1.588	0.05966	0.05014	0.835	Plant hormone
*T1_Unigene30289*	13.5	*T2_Unigene25344*	26.8	*At5G58130*	ROS3 protein REPRESSOR OF SILENCING 3	417	0.00511	0.00783	1.534	0.30256	0.26601	0.848	Transcriptional gene silence
*T1_Unigene25303*	11.4	*T2_Unigene21895*	11.8	*At5G63910*	FCLY farnesylcysteine lyase	789	0.00456	0.00687	1.509	0.04333	0.05374	1.25	
*T1_Unigene24651*	13.7	*T2_Unigene25310*	12.7	*At1G51600*	ZML2 GATA transcription factor 28	867	0.00338	0.00498	1.473	0.05834	0.04702	0.8	
*T1_Unigene24978*	27.5	*T2_Unigene23451*	42.2	*At2G15290*	Translocon at inner membrane of chloroplasts 21 (TIC21)	738	0.00527	0.00547	1.037	0.0501	0.05744	1.152	
*T1_Unigene31300*	25.4	*T2_Unigene16505*	32.9	*At3G03640*	BGLU25 beta glucosidase 25	1224	0.01055	0.01064	1.009	0.05242	0.05487	1.049	Biosynthesis of secondary metabolites

**FIGURE 6:**
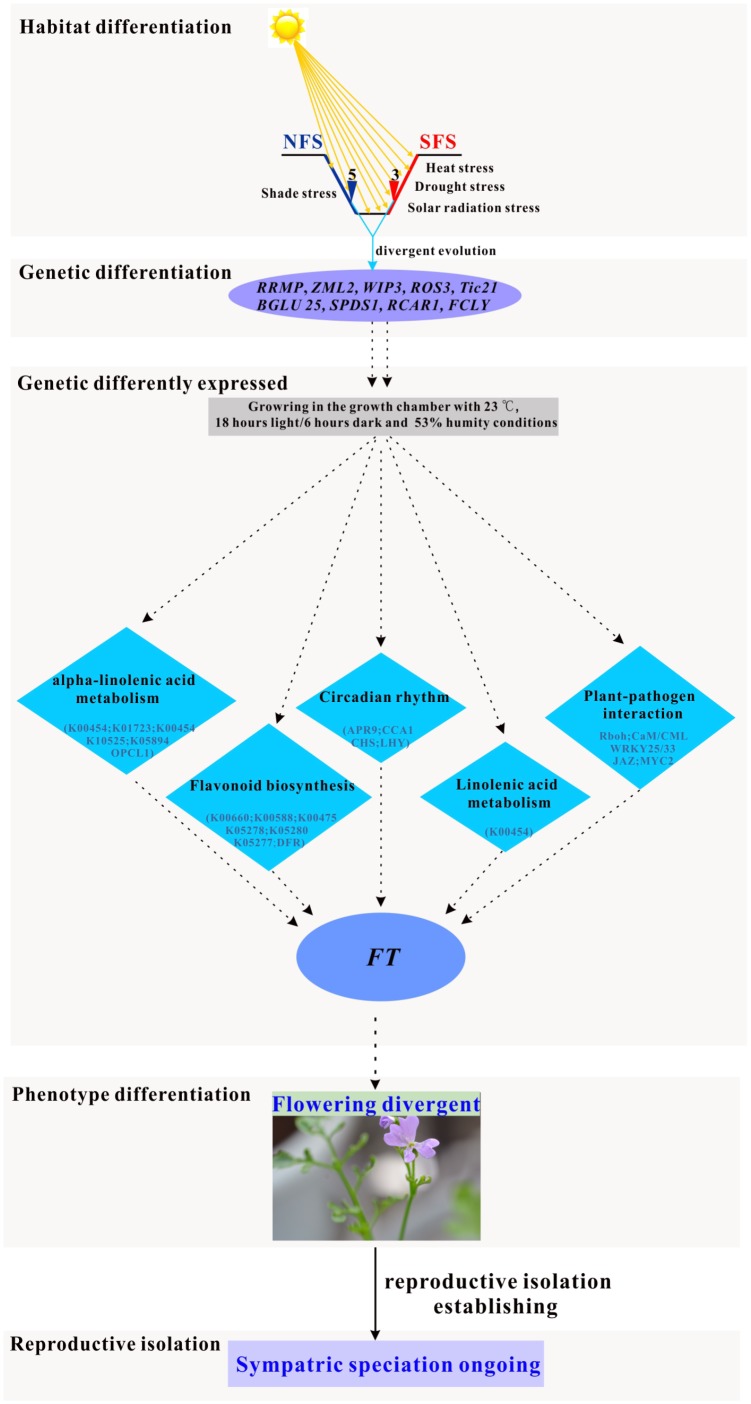
Simulated pathway of the ongoing sympatric speciation model in the two ecotypes of *R. lunaria* living on the opposite slopes of EC I.

Noteworthy, four of the isogenes (*spermidine synthase 1, farnesylcysteine lyase, Translocon at inner membrane of chloroplasts 21*, and *BGLU25 beta glucosidase 25*) also showed significant divergence between *R. lunaria* and *A. thaliana* with the *K*a/*K*s ratio over 1. *spermidine synthase 1* (*SPDS1*), a member of the spermidine synthase-related gene family that takes part in the polyamine biosynthesis, is responsible for spermidine accumulation in many plants and expressed in all plant organs. It plays an important role in the regulation of spermidine synthase activity, and is involved in different growth stages by modulating the contents of polyamines in plant cells (Hanzawa et al., 2002; Neily et al., 2011). *farnesylcysteine lyase* (*FCLY*), encoding a specific farnesylcysteine (FC) lyase, expresses in all *Arabidopsis* tissues and organs. It is proved to be involved in the recycling and oxidative metabolism of FC to farnesal and cysteine, and in the regulation of ABA signaling and meristem development by regulating the accumulation of FC and inhibition of isoprenylcysteine methyltransferase (ICMT) (Crowell et al., 2007; Huizinga et al., 2010). *translocon at inner membrane of chloroplasts 21* (*Tic 21*), also called *permease in chloroplasts1* (*PIC1*), acts as an essential part in protein translocation across the inner envelope membrane of chloroplast and regulates plant growth and development by directing homeostasis and transports of iron. It also plays a critical role in leaf development in the later stages (Teng et al., 2006; Duy et al., 2007, 2011; Lopezmillan et al., 2016). *beta glucosidase 25* (*BGLU25*) is involved in the carbohydrate metabolism and glycosyl compound metabolic process, which plays an important role in the regulation of hydrolyzing *O*-glycosyl compounds and activity of the beta-glucosidase and hydrolase (Xu et al., 2004). Considering that all of these four genes were also involved in the local adaptation between the two ecotypes of *R. lunaria*, we hypothesize that these genes could play important roles in plant speciation, at least in the Brassicaceae, and may be involved in the interslope sympatric speciation of *R. lunaria* in Evolution Canyon I. This phenomenon was also examined in Evolution Canyon II, as was done for *Bacillus simplex* (Sikorski and Nevo, 2005).

### Phenotypic Variations and Regulation Differentiation of Flowering Time Between the Two Ecotypes of *R. lunaria*

Under long day conditions (with 18 h light with 6 h dark), we found out that the ecotype of SFS3, which is adapted to the dry tropical biome, is flowering at least 3 months earlier than that of the humid temperate ecotype NFS5, as evidenced by 57 days for ecotype SFS3, while 149 days for ecotype NFS5. This dramatic earlier phase transition from nutrition growth to reproductive growth under drought stress was widely found in many other natural plant species, such as in *A. thaniana, Lupinus luteus* L. and wheat (Franks et al., 2007; Berger and Ludwig, 2014; Farooq et al., 2014; Kenney et al., 2014). It was also documented that, to avoid the heat stress, plants tend to flower earlier (Suter and Widmer, 2013; Abouelwafa and Amein, 2016; Ishimaru et al., 2016). Nevo et al. (2012) showed that ∼10 days earlier flowering in 10 wild barley, *Hordeum spontaneum*, and 10 wild emmer wheat, *Triticum dicoccoides*, populations across Israel, due to global warming between 1980 and 2008, highly support that earlier flowering is an effective way for plants to escape from both the drought and heat stresses.

Previous studies displayed by tiling array hybridizations on the wild sampling from the opposite slopes of EC I showed that DEGs were involved in at least the blue light signaling pathway, circadian rhythm and protein amino acid phosphorylation in response to the drought stress on the SFS. Interestingly, more heat-response genes are activated and a fivefold increase in flower development was recorded in the SFS ecotypes of *R. lunaria* (Brodsky et al., 2008). Although the circadian rhythm – plant pathway is not significantly differently expressed between the two ecotypes after genomic abundance correction, four up-regulated genes encoding three key enzymes were still detected between them, namely, *T1_Unigene26319* (*APR9*); *T1_Unigene31423* and *T2_Unigene30678* (*LHY*); *T2_Unigene24716* (*CCA1*) (Supplementary Figure S5). Until now, functions of these core circadian clock genes in plant’ stress response are still unclear. In this study, our controlled common garden experiments in the growth chamber witnessed both huge phenotypic (mainly on flowering time) and genomic divergence between the two interslope ecotypes. From our data, we found out that nine adaptive genes were involved in the post-transcriptional regulation, ABA-dependent response, photoprotective response, chloroplast protein-conducting channel and carbohydrate metabolic pathways, which might play a role in sensing and responding to the aforementioned multiple stresses. The huge divergence on their sequences between the two ecotypes indicated that they might have been also diversified with allelic functions, which were favored by the microecologically climatic heterogeneity of the interslopes during the sympatric speciation.

Considering that the expression patterns were different between the two ecotypes, both in wild and growth chamber conditions, the two ecotypes might have evolved divergent regulation systems responding to multiple stresses. In the model plant *Arabidopsis*, the flowering time was orchestrated by many pathways, including the photoperiodic pathway, vernalization pathway, autonomous pathway, and gibberellins (GA) pathway (Boss et al., 2004; Mulekar and Huq, 2012; Osnato et al., 2012; Kemi et al., 2013). As a close relative species of *Arabidopsis*, the regulation of flowering time in *R. lunaria* could be similar to the *Arabidopsis* species. Although plenty of studies supported that drought and heat stresses could promote the flowering time in plants (Franks et al., 2007; Suter and Widmer, 2013; Berger and Ludwig, 2014; Farooq et al., 2014; Kenney et al., 2014; Abouelwafa and Amein, 2016; Ishimaru et al., 2016), the crosstalk between modules is still unclear. In this study, we found out that the *ZML2* gene was under divergence selection, which could be integrated into the photoreceptor CRY1 responding to the photoprotection (Shaikhali et al., 2012). As *CRY1* is a well-known upstream geen involved in flowering time regulation (Yuan et al., 2016), we propose that *ZML2* might also play an important role in the differentiation of the flowering time between the two ecotypes as a consequence of local interslope adaptation. Thus, there might be some new pathways involved in the regulation of flowering time in response to the multiple stresses (Figure 6). After long-term local adaptation to the interslope microclimatically contrasting environments, both the phenotype traits and genomes of these two ecotypes of *R. lunaria* have already greatly diverged from each other, and further induced the differentiation of expression patterns of numerous genes. Under the same common garden environmental conditions in the growth chamber, the stresses would be different for each ecotype. For example, the conditions in the growth chamber could be shade stress for the SFS3 ecotype, while it could be high light stress for the NFS5 ecotype. To respond to multiple stresses, the common genes and pathways involved in biotic and abiotic stresses significantly differentially expressed multiple genes between the two ecotypes, which might consequently impact the expression patterns of the down-stream flowering time genes such as *FT1* (*FLOWERING LOCUS T1*). As a long evolutionary outcome, the flowering time of the two ecotypes have dramatically diverged, and further developed reproductive isolation. Due to the diversifying selection overruling gene flow (Nevo, 2011), this kind of incipient sympatrical speciation between the two ecotypes in Evolution Canyon I was also found in other species, such as wild barley (Nevo, 2006) and *Drosophila melanogaster* (Korol et al., 2006; Pavlíèek et al., 2008; Nevo, 2011). Of course, more genetic evidences are still needed to verify the molecular functions of these adaptive alleles in *R. lunaria*.

## Conclusion

Sympatric speciation is a common model widely existing across the canyons with climatic, geologic, and biotic differentiation between slopes. In this study, we fully investigated a new model for the incipient sympatric speciation at Evolution Canyon (Nevo, 2011). Based on the phenotypic investigation of flowering time in the common garden, we identified 3 months earlier flowering time of the dry tropical sun population of *R. lunaria* than the humid-cool temperate shade population. Likewise, between the opposite slope ecotypes, a total of 1,064 isogenic genes were significantly differentially expressed and nine genes significantly diverged that displayed specific interslope genetic-ecological adaptations. Both the flowering time and the expression patterns of the genes related to multiple stresses significantly differed between the two ecotypes. Clearly, flowering time is a cardinal variable of reproductive isolation, as a consequence of the genomic divergence between plant populations. This work substantiated a new species to the increasing list of either incipiently or fully sympatrically evolving species in Evolution Canyon.

As the other models in Evolution Canyon (Sikorski and Nevo, 2005; Nevo, 1995, 2014). Evolution Plateau (Hadid et al., 2013; Li K. et al., 2015, 2016; Zhao et al., 2016) and Evolution Slope (Wang et al., 2018), sympatric speciation of *R. lunaria* at Evolution Canyon I in Mount Carmel could also be tested at Evolution II in western Upper Galilee, which are separated by 40 km from EC I. Future projects of *R. lunaria* at both EC I and EC II could include intraslope and interslope crosses, investigations on genomics, transcriptomics, epigenomics, and metabolomics, which could shed light on the complexities of sympatric speciation.

## Author Contributions

X-FM and EN conceived the ideas and designed the investigations. CQ, XYa, HY, and X-FM analyzed the data and wrote the first version of the manuscript. CQ and HY collected the data. XF, XYa, and PS assisted in performing the experiments. ZL and EN provided improvements to the manuscript.

## Conflict of Interest Statement

The authors declare that the research was conducted in the absence of any commercial or financial relationships that could be construed as a potential conflict of interest.

## Footnotes

1 http://soap.genomics.org.cn/soapsnp.html

2 www.arabidopsis.org
