# Supplementary material for: Transcriptomes Divergence of Ricotia lunaria Between the Two Micro-Climatic Divergent Slopes at “Evolution Canyon” I, Israel
Source: Front Genet. 2018 Nov 14;9:506. doi: 10.3389/fgene.2018.00506 (PMC6246625; doi:10.3389/fgene.2018.00506)
Supplement: Supplementary file 1 [file Data_Sheet_1.docx]

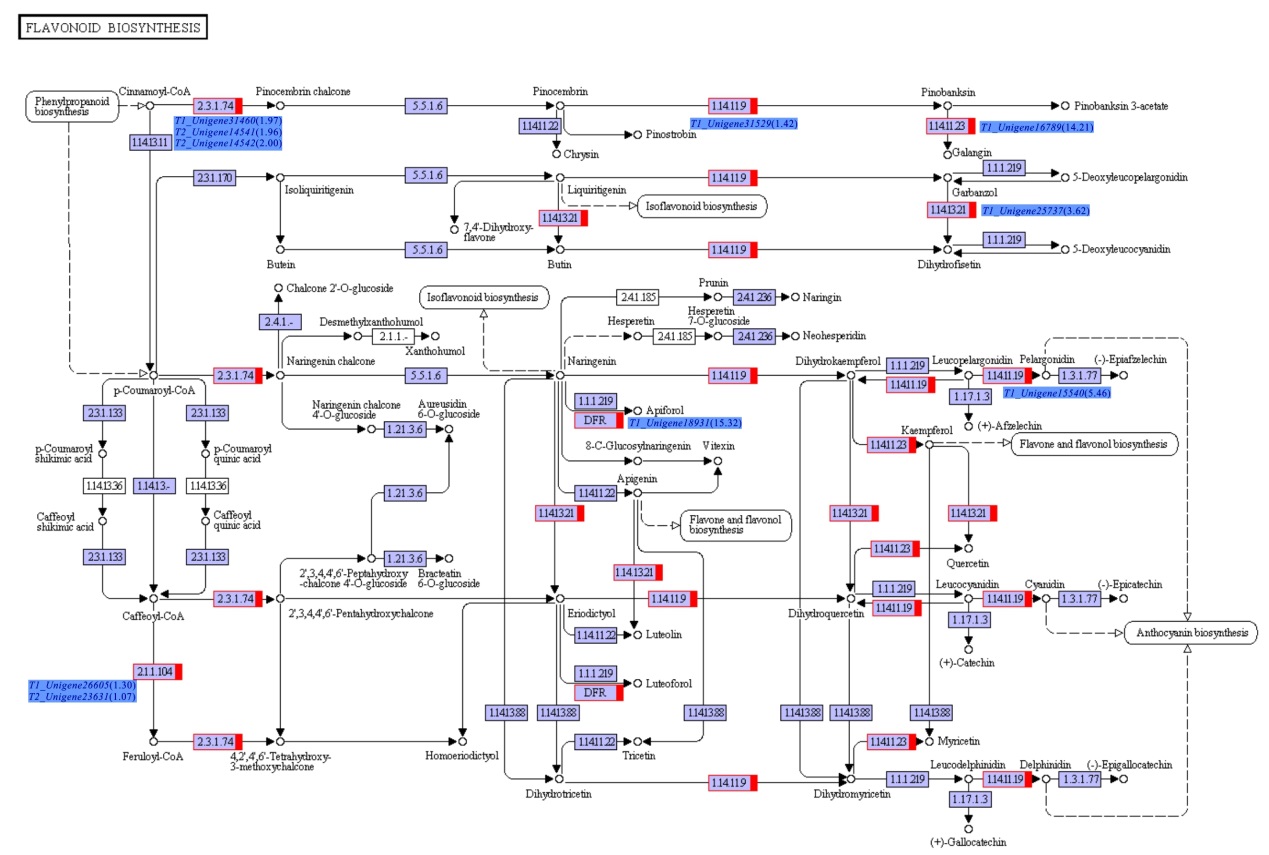


Fig. S1 Differential expression of flavonoid biosynthesis pathway implemented with KEGG pathway analysis. Enzymes with red box were up-regulated in either one of the two ecotypes.


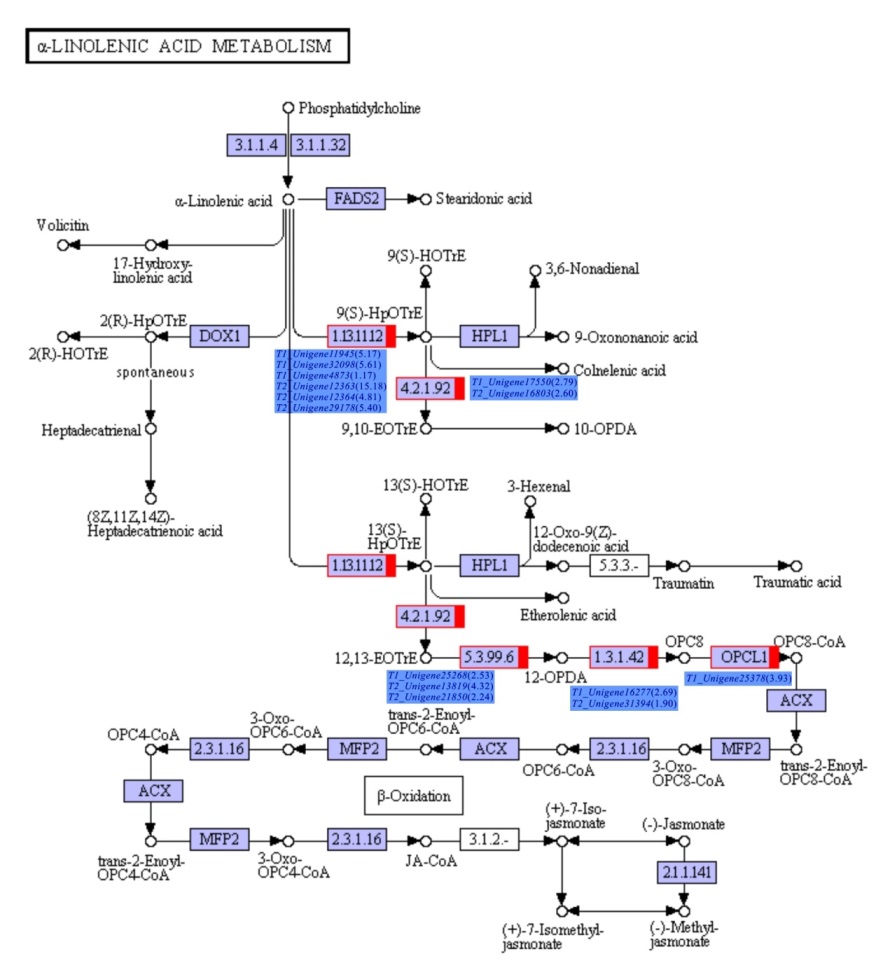


Fig. S2 Differential expression of *α*-linolenic acid metabolism pathway implemented with KEGG pathway analysis. Enzymes with red box were up-regulated in either one of the two ecotypes.


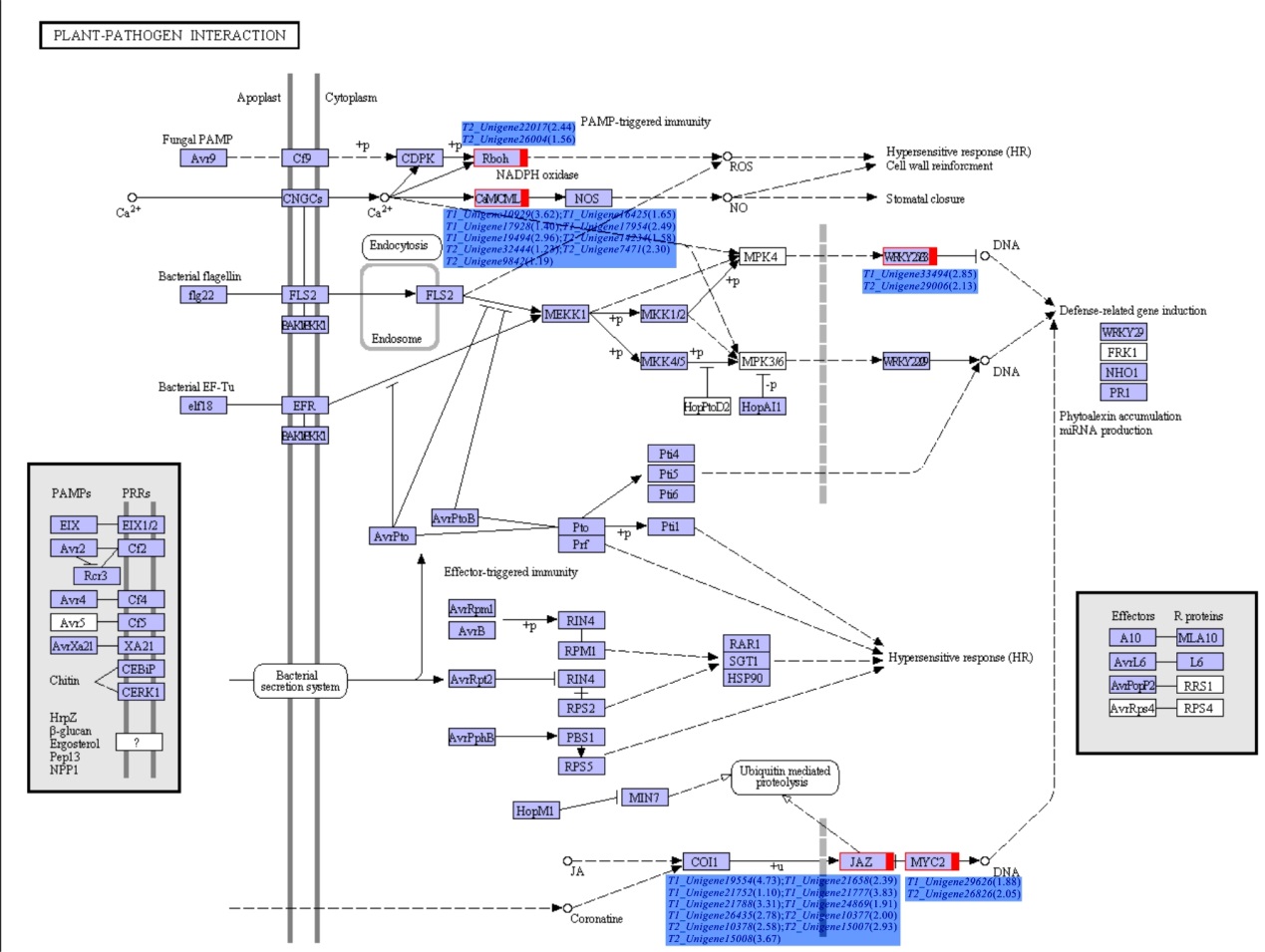


Fig. S3 Differential expression of plant-pathogen interaction pathway implemented with KEGG pathway analysis. Enzymes with red box were up-regulated in either one of the two ecotypes.


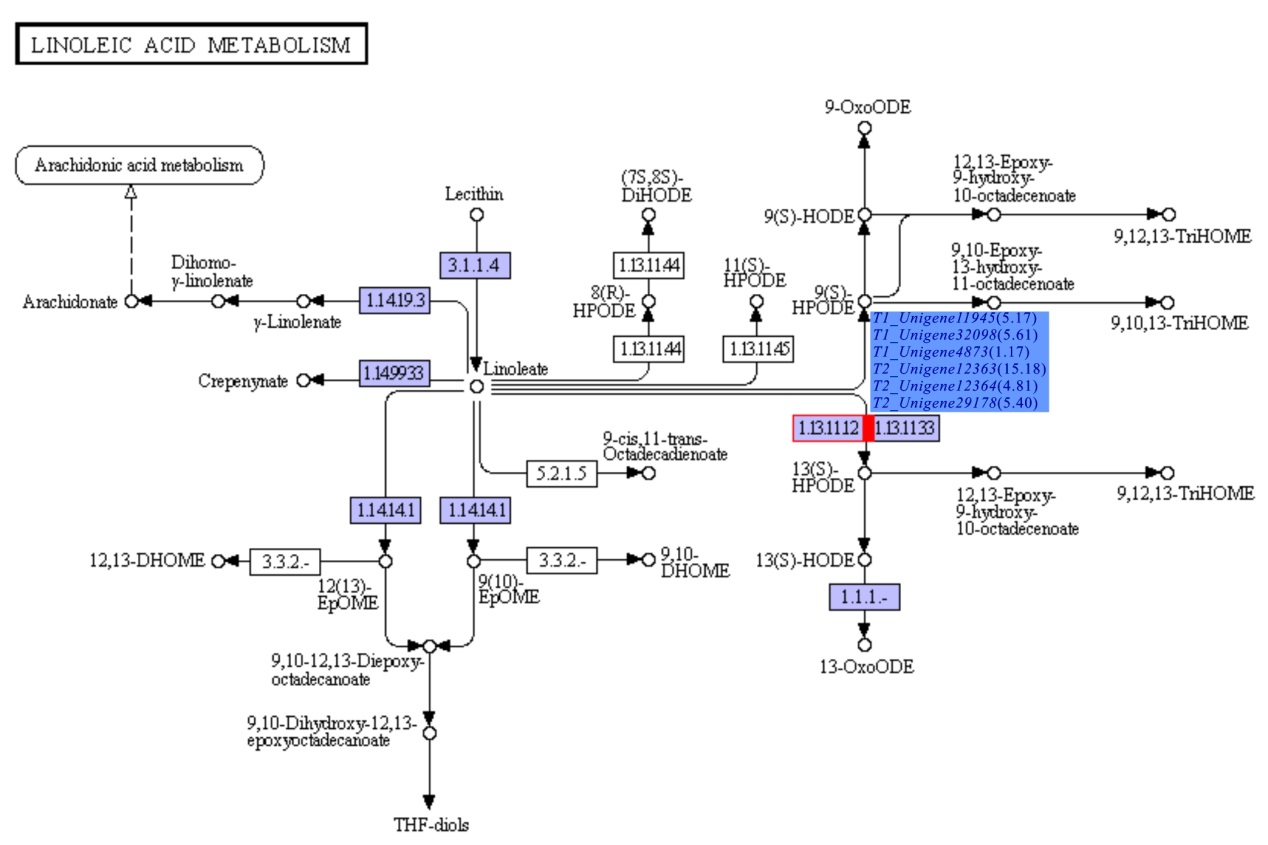


Fig. S4 Differential expression of linoleic acid metabolism pathway implemented with KEGG pathway analysis. Enzymes with red box were up-regulated in either one of the two ecotypes.


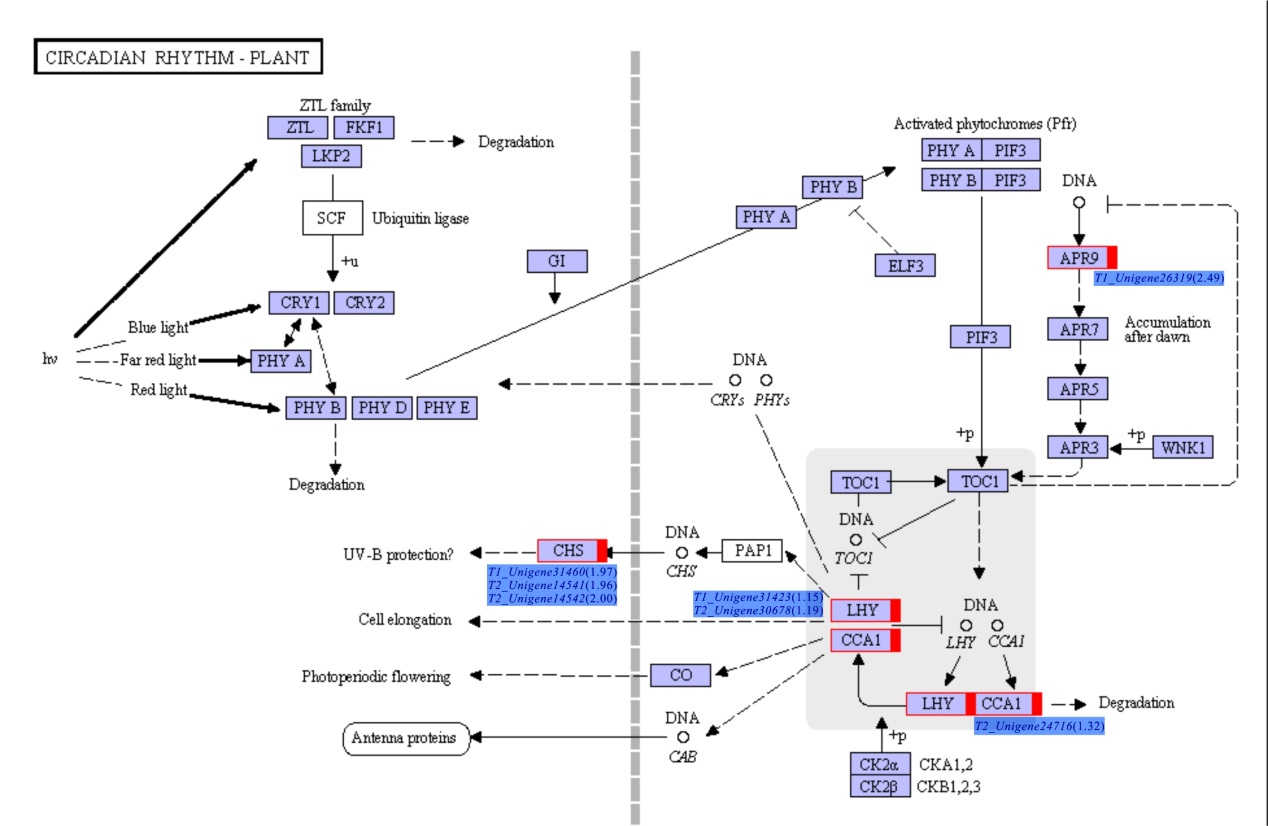


Fig. S5 Differential expression of circadian rhythm – plant pathway implemented with KEGG pathway analysis. Enzymes with red box were up-regulated in either one of the two ecotypes.

Table S3 Significantly differently expressed pathways in KEGG.

| KEGG_pathway | ko_id | F cluster* | F genome $ | P-value | Corrected P-value |
| --- | --- | --- | --- | --- | --- |
| Flavonoid biosynthesis | ko00941 | 10(3.3%) | 25(0.4%) | 9.77E-08 | 7.13E-06 |
| alpha-Linolenic acid metabolism | ko00592 | 14(4.6%) | 56(0.9%) | 2.43E-07 | 1.77E-05 |
| Plant-pathogen interaction | ko04626 | 27(8.9%) | 215(3.4%) | 3.54E-06 | 0.000259 |
| Linoleic acid metabolism | ko00591 | 6(2.0%) | 19(0.3%) | 0.000187 | 0.013667 |
| Circadian rhythm - plant | ko04712 | 7(2.3%) | 47(0.7%) | 0.00664 | 0.484741 |
| Phenylalanine metabolism | ko00360 | 11(3.6%) | 105(1.7%) | 0.011661 | 0.851267 |
| Plant hormone signal transduction | ko04075 | 25(8.2%) | 325(5.2%) | 0.012783 | 0.933194 |

Note: * frequency in the all 303 KEGG clusters differentiated expressed; $ frequency in the all 6303 KEGG pathways annotated in the all unigenes.

Table S4 DEGs involved into the GO terms of biological process.

| Gene_Ontology_term | GO | Clusters | Genome | *P*-value | Corrected  *P*-value |
| --- | --- | --- | --- | --- | --- |
| jasmonic acid biosynthetic process | GO:0009695 | 79 | 498 | 0 | 0 |
| response to chitin | GO:0010200 | 170 | 1989 | 0 | 0 |
| response to fungus | GO:0009620 | 58 | 376 | 0 | 0 |
| response to jasmonic acid stimulus | GO:0009753 | 90 | 966 | 0 | 0 |
| response to mechanical stimulus | GO:0009612 | 41 | 247 | 0 | 0 |
| abscisic acid mediated signaling pathway | GO:0009738 | 101 | 1024 | 0 | 0 |
| response to water deprivation | GO:0009414 | 134 | 1520 | 0 | 0 |
| hyperosmotic salinity response | GO:0042538 | 104 | 905 | 0 | 0 |
| sexual reproduction | GO:0019953 | 23 | 87 | 6.76E-13 | 1.21E-09 |
| response to cold | GO:0009409 | 143 | 1747 | 1.36E-11 | 2.44E-08 |
| ethylene mediated signaling pathway | GO:0009873 | 60 | 551 | 1.80E-11 | 3.22E-08 |
| response to wounding | GO:0009611 | 182 | 1625 | 2.87E-11 | 5.12E-08 |
| jasmonic acid mediated signaling pathway | GO:0009867 | 108 | 1313 | 3.79E-11 | 6.76E-08 |
| response to absence of light | GO:0009646 | 20 | 81 | 9.61E-11 | 1.72E-07 |
| defense response to fungus | GO:0050832 | 138 | 1875 | 1.66E-10 | 2.96E-07 |
| jasmonic acid mediated signaling pathway | GO:0009864 | 20 | 86 | 3.08E-10 | 5.50E-07 |
| respiratory burst involved in defense response | GO:0002679 | 68 | 710 | 3.09E-10 | 5.51E-07 |
| response to auxin stimulus | GO:0009733 | 96 | 1163 | 3.50E-10 | 6.26E-07 |
| ethylene biosynthetic process | GO:0009693 | 46 | 398 | 6.64E-10 | 1.19E-06 |
| regulation of plant-type hypersensitive response | GO:0010363 | 132 | 1819 | 1.04E-09 | 1.86E-06 |
| cellular cation homeostasis | GO:0030003 | 45 | 390 | 1.06E-09 | 1.89E-06 |
| response to karrikin | GO:0080167 | 66 | 706 | 1.52E-09 | 2.72E-06 |
| response to ethylene stimulus | GO:0009723 | 84 | 1020 | 4.58E-09 | 8.18E-06 |
| protein targeting to membrane | GO:0006612 | 124 | 1770 | 2.37E-08 | 4.23E-05 |
| positive regulation of flavonoid biosynthetic process | GO:0009963 | 50 | 515 | 4.17E-08 | 7.45E-05 |
| response to sucrose stimulus | GO:0009744 | 73 | 900 | 8.06E-08 | 0.000144 |
| anthocyanin-containing compound biosynthetic process | GO:0009718 | 26 | 195 | 1.95E-07 | 0.000348 |
| intracellular signal transduction | GO:0035556 | 52 | 575 | 2.16E-07 | 0.000387 |
| divalent metal ion transport | GO:0070838 | 33 | 298 | 4.21E-07 | 0.000752 |
| response to desiccation | GO:0009269 | 26 | 211 | 9.11E-07 | 0.001628 |
| response to herbivore | GO:0080027 | 12 | 51 | 9.34E-07 | 0.001669 |
| pollen hydration | GO:0009859 | 5 | 7 | 2.44E-06 | 0.004362 |
| response to oxidative stress | GO:0006979 | 56 | 695 | 2.91E-06 | 0.005193 |
| negative regulation of programmed cell death | GO:0043069 | 75 | 1031 | 3.28E-06 | 0.005853 |
| cellular response to freezing | GO:0071497 | 12 | 58 | 4.00E-06 | 0.00715 |
| toxin catabolic process | GO:0009407 | 53 | 655 | 4.66E-06 | 0.008316 |
| defense response by callose deposition | GO:0052542 | 25 | 221 | 6.83E-06 | 0.0122 |
| defense response to bacterium | GO:0042742 | 113 | 1771 | 7.57E-06 | 0.01352 |
| regulation of nitric oxide metabolic process | GO:0080164 | 6 | 14 | 1.17E-05 | 0.020846 |
| response to UV-B | GO:0010224 | 40 | 465 | 1.61E-05 | 0.02879 |
| photosynthesis, light reaction | GO:0019684 | 24 | 218 | 1.63E-05 | 0.029054 |
| oxylipin biosynthetic process | GO:0031408 | 14 | 90 | 2.12E-05 | 0.037926 |
